# Association of thrombocytopenia with immune checkpoint inhibitors: a large-scale pharmacovigilance analysis based on the data from FDA adverse event reporting system database

**DOI:** 10.3389/fphar.2024.1407894

**Published:** 2024-06-17

**Authors:** Geliang Liu, Shuxian Zhang, Zhuang Mo, Tai Huang, Qi Yu, Xuechun Lu, Peifeng He

**Affiliations:** ^1^ Shanxi Key Laboratory of Big Data for Clinical Decision, Shanxi Medical University, Taiyuan, China; ^2^ Key Laboratory of Cellular Physiology, Ministry of Education, Shanxi Medical University, Taiyuan, China; ^3^ School of Management, Shanxi Medical University, Taiyuan, China; ^4^ School of Basic Medical Sciences, Shanxi Medical University, Taiyuan, China; ^5^ Department of Hematology, The Second Medical Center of the China PLA General Hospital and National Center for Clinical Medicine of Geriatric Diseases, Beijing, China; ^6^ Institute of Medical Data Sciences, Shanxi Medical University, Taiyuan, China

**Keywords:** immune checkpoint inhibitors, immune thrombocytopenia, immune-related adverse events, FAERS, TCGA

## Abstract

**Introduction:** An increasing number of immune-related adverse events (irAEs) induced by immune checkpoint inhibitors (ICIs) have been reported during clinical treatment. We aimed to explore the clinical characteristics of patients with ICIs-induced ITP under different therapeutic strategies based on the FAERS database and explore the potential biological mechanisms in combination with TCGA pan-cancer data.

**Methods:** Data from FAERS were collected for ICIs adverse reactions between January 2012 and December 2022. Disproportionality analysis identified ICIs-induced ITP in the FAERS database using the reporting odds ratio (ROR), proportional reporting ratio (PRP), Bayesian confidence propagation neural network (BCPNN), and multi-item gamma Poisson shrinker algorithms (MGPS). The potential biological mechanisms underlying ITP induced by ICIs were examined using TCGA transcriptome data on cancers.

**Results:** In the FAERS, 345 ICIs-induced ITP reports were retrieved, wherein 290 (84.06%) and 55 (15.94%) were reported as monotherapy and combination therapy, respectively. The median age of the reported patients with ICIs-induced ITP was 69 years (IQR 60-76), of which 62 (18%) died and 47 (13.6%) had a life-threatening outcome. The majority of reported indications were lung, skin, and bladder cancers, and the median time to ITP after dosing was 42 days (IQR 17-135), with 64 patients (43.5%) experiencing ITP within 30 days of dosing and 88 patients experiencing ITP in less than 2 months (59.9%). The occurrence of ICIs-induced ITP may be associated with ICIs-induced dysregulation of the mTORC1 signaling pathway and megakaryocyte dysfunction.

**Conclusion:** There were significant reporting signals for ITP with nivolumab, pembrolizumab, cemiplimab, atezolizumab, avelumab, durvalumab, ipilimumab, nivolumab/ipilimumab, and pembrolizumab/ipilimumab. Patients treated with anti-PD-1 in combination with anti-CTLA-4 are more likely to have an increased risk of ICIs-induced ITP. Patients with melanoma are at a higher risk of developing ITP when treated with ICI and should be closely monitored for this risk within 60 days of treatment. The potential biological mechanism of ICIs-induced ITP may be related to the dysfunction of megakaryocyte autophagy through the overactivation of the mTOR-related signaling pathway. This study provides a comprehensive understanding of ICIs-induced ITP. Clinicians should pay attention to this potentially fatal adverse reaction.

## 1 Introduction

Globally, tumors continue to be a significant contributor to human mortality. Over the past decade, the discovery of immune checkpoint inhibitors (ICIs) has revolutionized the treatment of patients with tumors. ICIs, including programmed cell death protein-1 (PD-1), programmed death-ligand 1 (PD-L1), and cytotoxic T cell-associated protein-4 (CTLA-4), kill tumor cells by activating the immune system (Marei et al., 2023). Currently, ICIs are approved for treating a broad spectrum of malignancies. Although they can largely improve the prognosis of patients with tumors, they can also lead to serious adverse events, known as immune-related adverse events (irAEs), which indicate that the patient’s autoimmune system is overactivated and can involve most organs, including the skin, gastrointestinal tract, heart, kidneys, and liver. These irAEs are the main reasons for treatment discontinuation, and even relatively mild irAEs can greatly deteriorate the quality of life of patients, whereas severe irAEs can lead to death (Ramos-Casals et al., 2020).

With the extensive use of ICIs in oncology therapy, rare irAEs have emerged, including hematological immune-related adverse events (hem-irAEs). Hem-irAEs primarily include aplastic anemia, immune thrombocytopenia (ITP), and autoimmune hemolytic anemia. Although ICIs-induced ITP is relatively rare, it can be life-threatening in severe cases (Li et al., 2019). ITP is an autoimmune disease characterized by T-cell dysregulation, which is characterized by the production of autoantibodies against platelet antigens. Immune cells mistakenly attack platelets, resulting in frequent bruising and unstoppable bleeding. Once ITP occurs, it requires temporary termination with antitumor therapy if the condition grade is ≥3, and it may even be life-threatening in severe cases; therefore, clinicians should be vigilant and monitor for indicators of this potentially serious irAEs (Kroll et al., 2022). However, ICIs-induced ITP has not been extensively studied, and little is known about its onset, indications, clinical features, and biological mechanisms. Therefore, in this study, we retrieved ICIs-induced ITP data based on all irAEs between 2012 and 2022 from the U.S. Food and Drug Administration (FDA) Adverse Event Reporting System (FAERS) database, performed disproportionality analysis, and explored the potential biological mechanisms associated with ICIs-induced ITP in combination with The Cancer Genome Atlas (TCGA) data.

## 2 Methods

### 2.1 Data source

The pharmacovigilance data used in this study were obtained from the FAERS database, which monitors adverse events in medicines and therapeutic procedures by collecting adverse drug reaction reports submitted by healthcare professionals and consumers. Healthcare professionals in the FAERS database include physicians, pharmacists, and other healthcare professional. These reports cover a broad spectrum of adverse reactions during drug therapy, ranging from minor discomfort to serious adverse reactions, and contain data on clinical characteristics such as patient demographics, indications, and survival outcomes. We obtained all adverse reaction reports from the FAERS database from January 2012 to December 2022 for subsequent analyses. Adverse events for subsequent analyses were based on the preferred terms in the Medical Dictionary for Regulatory Activities (MedDRA) (version 25.0). The preferred term “immune thrombocytopenia” was used to identify ICIs-induced ITP cases. In addition, the transcriptomic data used for pan-cancer association analysis in this study were obtained from TCGA, which is a project co-sponsored by the National Institutes of Health and National Cancer Institute that contains multi-omics data on dozens of cancers, including breast, colorectal, and lung cancers, and aims to systematically study the genomes of human cancers to reveal the complex mechanisms of cancer development (Cancer Genome Atlas Research Network et al., 2013).

### 2.2 Data processing procedure

**FIGURE 1:**
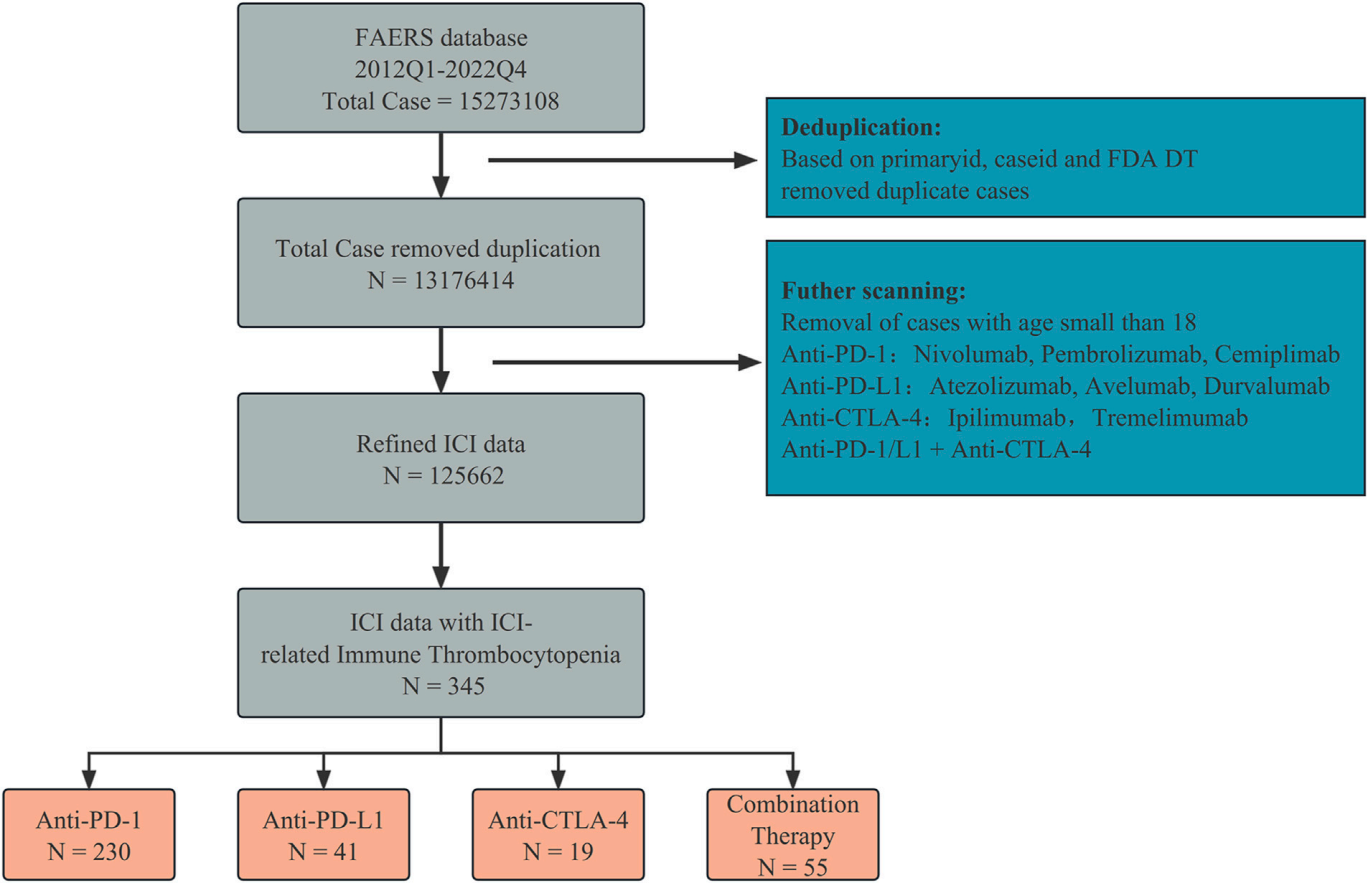
Data filtering process of FAERS performed in this study.

We screened the data collected from the FAERS database (Figure 1). The drugs used were anti-PD-1 (nivolumab, pembrolizumab, and cemiplimab), anti-PD-L1 (atezolizumab, avelumab, and durvalumab), and anti-CTLA-4 (ipilimumab and tremelimumab). Duplicate reports were excluded based on the PRIMARYID, CASEID, and FDA DT fields. We retained only cases and adverse reaction reports of patients aged over 18 years, and only anti-PD-1/L1 + anti-CTLA-4 was considered as a combination therapy strategy. To eliminate bias introduced by different reporters, we corrected for indications and medications taken as indicated in the acquired reports. Case reports of ICIs-induced ITP adverse reactions (N = 345) were retrieved for further analysis.

### 2.3 Signal mining

Disproportionality analysis was performed on the retrieved case reports by applying four different signal detection algorithms to detect associations between ICI treatment strategies and ITP events. The aforementioned four algorithm formulas and threshold criteria are listed in Table 1 (Guo et al., 2023). In this study, all four threshold criteria had to be met to produce a positive signal for an ITP adverse event. When performing a joint analysis with the TCGA data, we calculated only the ROR (Zhou et al., 2023). In addition, we performed descriptive analyses of the case reports for different ICI treatment strategies, including sex, age, time to onset, country and type of person reporting, year of FDA receipt of the report, outcome events, and indications for treatment. Patient outcomes included death, life-threatening events, hospitalization, and disability. The time to onset was calculated as the time to treatment initiation minus the time to adverse events.

**TABLE 1:** Summary of algorithms used for signal detection.

Algorithms	Equation	Threshold value
ROR	ROR=ad/bc	N ≥ 2, 95%CI > 1
	95%CI=elnROR±1.961/a+1/b+1/c+1/d^0.5	
PRR	$\text{PRR}=a\left(c+d\right)/c/\left(a+b\right)$	N ≥ 2, PRR ≥2, χ^2^ ≥ 4
	χ2=ad‐bc^2a+b+c+d/a+bc+da+cb+d	
BCPNN	$\text{IC}=\log_{2}^{a\left(a+b+c+d\right)\left(a+c\right)\left(a+b\right)}$	IC025 > 0
	$\text{IC}025=e^{\left(\ln\;\left(\text{IC}\right)-1.96(1/a+1/b+1/c+1/d\hat{)}0.5\right)}$	
MGPS	$\text{EBGM}=a\left(a+b+c+d\right)/\left(a+c\right)/\left(a+b\right)$	EBGM05 > 2
	$\text{EBGM}05=e^{\left(\ln\;\left(\text{EBGM}\right)-1.64\left(1/a+1/b+1/c+1/d\hat{)}0.5\right.\right)}$	

### 2.4 Pan-cancer analysis in combination with TCGA

We downloaded transcriptome data in FPKM format from TCGA database for 28 tumor classes and subsequently converted them to the TPM format. The Kyoto Encyclopedia of Genes and Genomes (KEGG) and Reactome pathway gene sets were obtained from the MsigDB database, and all tumor transcriptome data were analyzed by single-sample gene set enrichment analysis (ssGSEA) using the gsva package in R. Thus, the enrichment scores of different tumor types in different signaling pathways were calculated (Subramanian et al., 2005). In R, the xCell package was used to calculate the enrichment scores for 64 immune and stromal cells from various types of tumors (Aran et al., 2017). To identify the biological mechanisms underlying ICIs-induced ITP, we will analyze the association between the ICIs-induced ITP ROR and the enrichment score of biological pathways and stromal cells at the pan-cancer level.

### 2.5 Statistical analysis

Chi-square tests were used to determine whether categorical variables were associated with mortality. We used the Kaplan-Meier method to estimate event-free probabilities for the time to onset of ICIs-induced ITP. Time to onset was compared between the groups using the log-rank test. Spearman’s correlation test was used to analyze the correlation between the ICIs-induced ITP ROR and the activation levels of biological pathways and immune and stromal cells at the pan-cancer level. Statistical significance was defined as *p* < 0.05. All statistical analyses and visualizations were performed using R (https://www.r-project.org/, version 4.2.1) and ChiPlot (https://www.chiplot.online/).

## 3 Results

### 3.1 Overall ICIs-induced ITP in the FAERS database, 2012—2022

**FIGURE 2:**
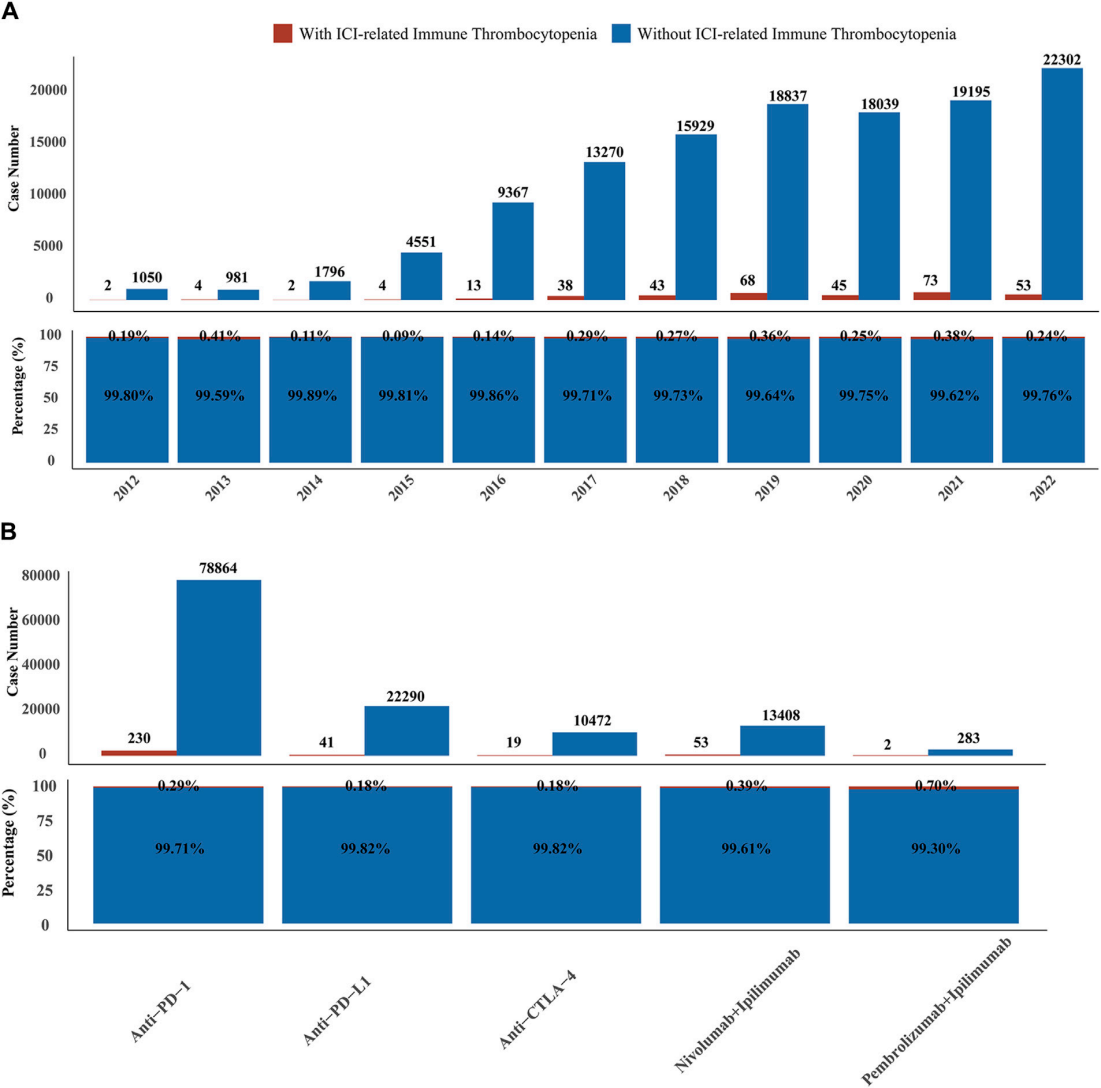
Overview of data on adverse events associated with ICIs-induced ITP from the FAERS database from 2012 to 2022. **(A)** The upper bar chart indicates the number of ICIs-induced and non-ICIs-induced ITP reports in the FAERS database from 2012 to 2022. The lower stacked graph shows the relative proportions of the ICIs-induced and non-ICIs-induced ITP reports in the FAERS database from 2012 to 2022. **(B)** The upper bar chart shows the number of ICIs-induced and non-ICIs-induced ITP reports under different ICI treatment strategies in the FAERS database from 2012 to 2022. The lower stacked graph shows the relative proportions of ICIs-induced and non-ICIs-induced ITP reports under different ICI treatment strategies in the FAERS database from 2012 to 2022.

We first obtained all reports of irAEs from the FAERS database (2012—2022) and retrieved reports of ICIs-induced ITP adverse events. ICIs-induced ITP accounted for a small proportion of reported irAEs, with 345 case reports, or 0.27% (345/125662) of the total reports. Overall, the year with the highest percentage of ICI-induced ITP cases was 2013 (0.41%). The lowest percentage occurred in year 2015 at 0.09% of the years, which was a very small but relatively stable percentage (Figure 2A). In terms of ICI treatment strategies, anti-PD-1-related ITP adverse reactions were reported in the highest number, accounting for 0.29% of the cases. When patients were treated with anti-PD-L1 or anti-CTLA4, the incidence of ITP adverse reactions was 0.18% in both cases. When patients were treated with a combination of anti-PD-1/L1 and anti-CTLA-4 antibodies, the incidence of ITP adverse events was 0.4% (Figure 2B). In conclusion, although rare, ICIs-induced ITP occurs relatively steadily under all treatment strategies for ICIs, and the proportion of ICIs-induced ITP increases significantly with combination therapy.

### 3.2 Descriptive analysis of cases with ICIs-induced ITP

**FIGURE 3:**
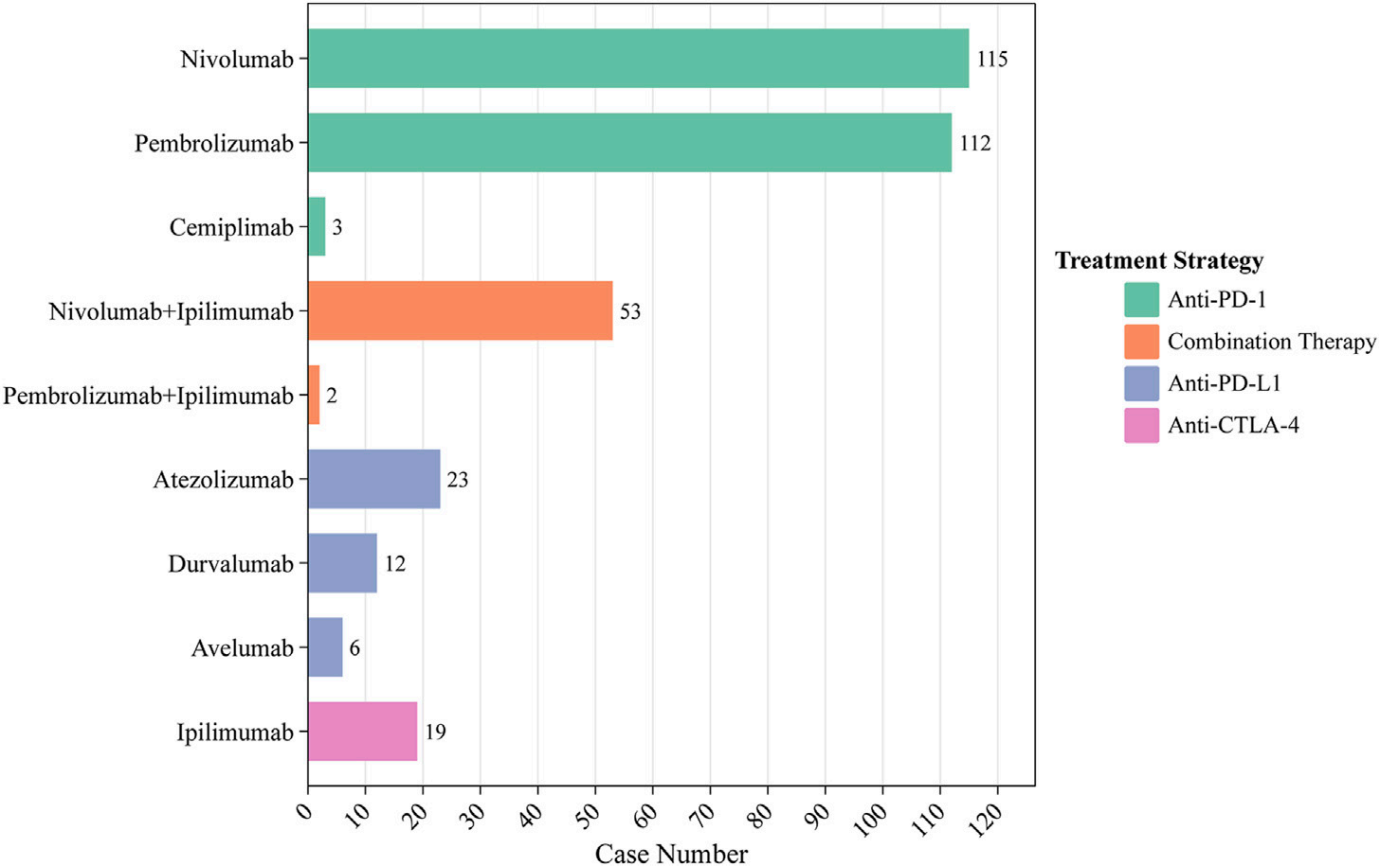
Number of ICIs-induced ITP reports for different ICI treatment strategies.

**FIGURE 4:**
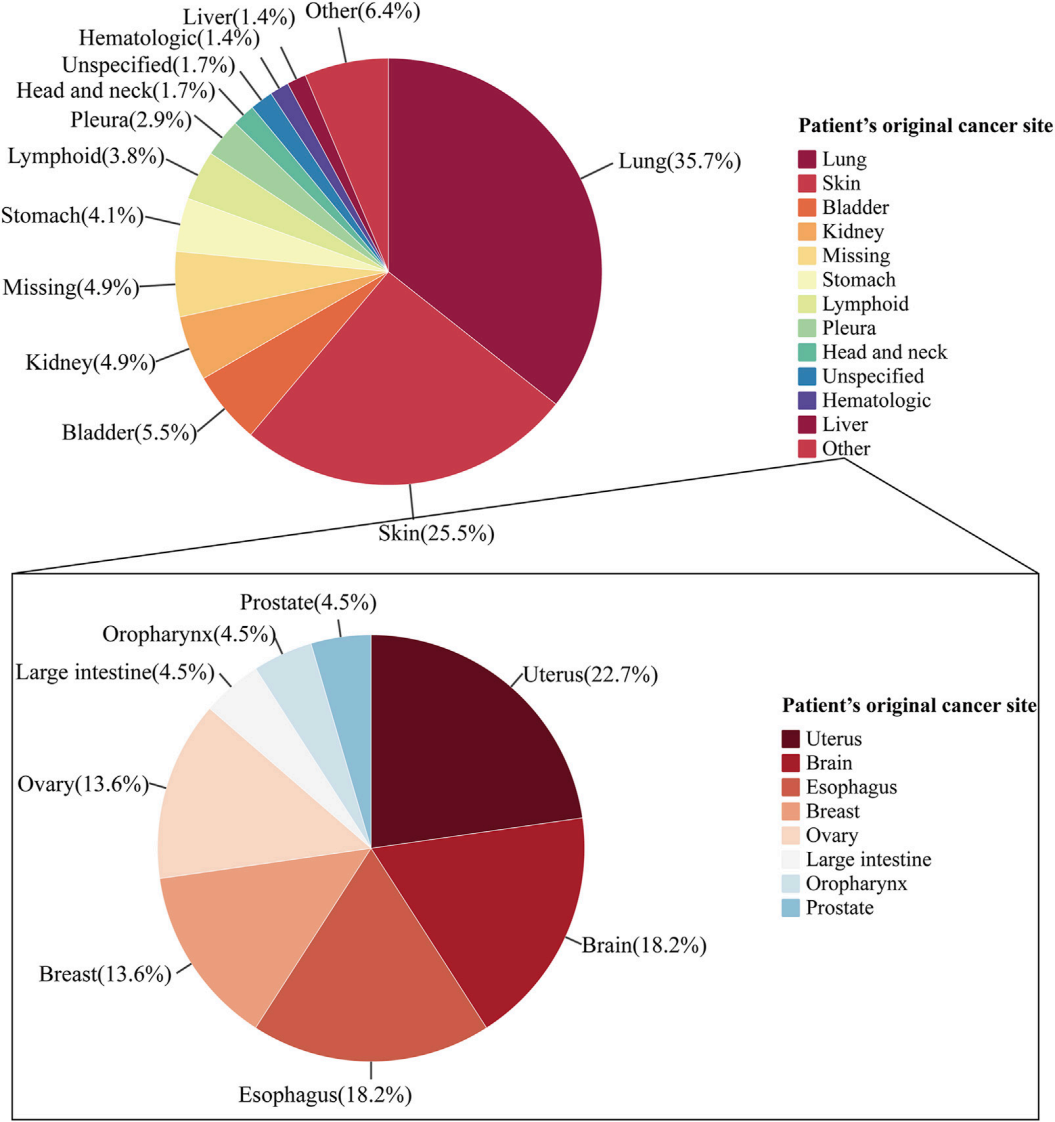
Pie chart of indications in ICIs-induced ITP reports. The pie chart shows the proportional composition of the patient’s cancer original sites. The pie chart on the top shows the proportional distribution of the case numbers for organs which were greater than 10. The pie chart at the bottom shows the proportional distribution of the case numbers for cancer originated from other.

Between 2012 and 2022, 345 ICIs-induced ITP cases were reported in the FAERS database. We first examined the ITP adverse reaction signals of patients, including those receiving seven ICI monotherapies and two ICI combination therapies, using four algorithmic criteria (Table 2). The results showed that all ICIs treatment regimens met the four criteria. Among them, pembrolizumab combined with ipilimumab had the highest ROR (14.9, 95%CI 3.7-59.9) for the treatment strategy. Whereas in monotherapy, avelumab had the highest ROR (14.6, 95%CI 6.5-32.5). Furthermore, we statistically analyzed these case reports (Table 3). The largest percentage was for anti-PD-1 (230/345, 66.7%), followed closely by combination therapy and anti-PD-L1 (combination therapy: 55/345, 15.9%; anti-PD-L1:41/345, 11.9%), while the smallest percentage was for anti-CTLA-4 (19/345, 5.5%) (Figure 3). In this study, patients were categorized into three groups using cutoff ages of 65 and 85 years, with a predominance of patients older than 65 years and younger than 85 years (177/345, 51.3%); the median age of the patients was 69 years (interquartile range [IQR] 60-76). The study found that death occurred in 18% of all patients and 13.6% experienced life-threatening outcomes. The indications accounting for most ICIs-induced ITP cases were lung (123/345, 35.7%), skin (88/345, 25.5%), and bladder cancers (19/345, 5.5%) (Figure 4). Most case reports were submitted by healthcare professionals (303/345, 87.8%). From a national perspective, the majority came from Japan (151/345, 43.8%), the United States (75/345, 21.7%), and France (24/345, 7.0%).

**TABLE 2:** Associations of different ICI treatment strategy with immune thrombocytopenia.

ICIs treatment strategy	N	ROR (95% CI)	PRR (χ^2^)	IC (IC025)	EBGM (EBGM05)
Total	345	8.3 (7.4,9.2)	8.3 (2051)	3.0 (2.7)	7.8 (7.1)
Anti-PD-1	230	7.4 (6.4,8.4)	7.4 (1,205)	2.8 (2.5)	7.1 (6.3)
Nivolumab	115	5.8 (4.8,7)	5.8 (448.7)	2.5 (2.1)	5.7 (4.9)
Pembrolizumab	112	9.4 (7.8,11.4)	9.4 (825.4)	3.2 (2.7)	9.2 (7.9)
Cemiplimab	3	7.0 (2.3,21.9)	7.0 (15.5)	2.8 (0.9)	7.0 (2.7)
Anti-PD-L1	41	6.0 (4.4,8.2)	6.0 (169.2)	2.6 (1.9)	6.0 (4.6)
Atezolizumab	23	5.0 (3.3,7.5)	5.0 (72.8)	2.3 (1.5)	5.0 (3.5)
Avelumab	6	14.6 (6.5,32.5)	14.6 (75.7)	3.9 (1.7)	14.5 (7.4)
Durvalumab	12	6.5 (3.7,11.5)	6.5 (56)	2.7 (1.5)	6.5 (4.1)
Ipilimumab (Anti-CTLA-4)	19	4.0 (2.6,6.3)	4.0 (43.1)	2.0 (1.3)	4.0 (2.8)
Nivolumab + ipilimumab	53	10.0 (7.6,13.1)	10.0 (424.4)	3.3 (2.5)	9.9 (7.9)
Pembrolizumab + ipilimumab	2	14.9 (3.7,59.9)	14.9 (26)	3.9 (1)	14.9 (4.7)

**TABLE 3:** Clinical characteristics of patients with immune checkpoint inhibitor-induced immune thrombocytopenia collected from the FAERS database (January 2012 to December 2022).

Clinical characteristics	Fatal (N = 62)	Non-fatal (N = 283)	Total (N = 345)	*p*-value
Gender				
Male	39 (62.9%)	172 (60.8%)	211 (61.2%)	0.806
Female	20 (32.3%)	78 (27.6%)	98 (28.4%)	
Missing	3 (4.8%)	33 (11.7%)	36 (10.4%)	
Age group				
18-65	21 (33.9%)	69 (24.4%)	90 (26.1%)	0.769
65-85	36 (58.1%)	141 (49.8%)	177 (51.3%)	
>85	1 (1.6%)	6 (2.1%)	7 (2.0%)	
Missing	4 (6.5%)	67 (23.7%)	71 (20.6%)	
Country				
JP	36 (58.1%)	115 (40.6%)	151 (43.8%)	0.001
US	3 (4.8%)	72 (25.4%)	75 (21.7%)	
FR	2 (3.2%)	22 (7.8%)	24 (7.0%)	
DE	5 (8.1%)	15 (5.3%)	20 (5.8%)	
CA	4 (6.5%)	5 (1.8%)	9 (2.6%)	
CN	1 (1.6%)	5 (1.8%)	10 (2.9%)	
Other country	11 (17.7%)	25 (8.8%)	36 (16.2%)	
Received year				
2012	0	2 (0.7%)	2 (0.6%)	0.742
2013	2 (3.2%)	2 (0.7%)	4 (1.2%)	
2014	0	2 (0.7%)	2 (0.6%)	
2015	0	4 (1.4%)	4 (1.2%)	
2016	1 (1.6%)	12 (4.2%)	13 (3.8%)	
2017	5 (8.1%)	33 (11.7%)	38 (11.0%)	
2018	7 (11.3%)	36 (12.7%)	43 (12.5%)	
2019	14 (22.6%)	54 (19.1%)	68 (19.7%)	
2020	8 (12.9%)	37 (13.1%)	45 (13.0%)	
2021	15 (24.2%)	58 (20.5%)	73 (21.2%)	
2022	10 (16.1%)	43 (15.2%)	53 (15.4%)	
Treatment strategy				
Anti-PD-1	42 (67.7%)	188 (66.4%)	230 (66.7%)	0.992
Anti-PD-L1	7 (11.3%)	34 (12.0%)	41 (11.9%)	
Anti-CTLA-4	3 (4.8%)	16 (5.7%)	19 (5.5%)	
Combination therapy	10 (16.1%)	45 (15.9%)	55 (15.9%)	
Outcomes				
Death	62 (100%)	0	62 (18.0%)	<0.001
Life-threatening	0	47 (16.6%)	47 (13.6%)	
Hospitalization	0	108 (38.2%)	108 (31.3%)	
Disability	0	1 (0.4%)	1 (0.3%)	
Other	0	126 (44.5%)	126 (36.5%)	
Missing	0	1 (0.4%)	1 (0.3%)	
Indication organ				
Lung	29 (46.8%)	94 (33.2%)	123 (35.7%)	0.166
Skin	13 (21.0%)	75 (26.5%)	88 (25.5%)	
Bladder	2 (3.2%)	17 (6.0%)	19 (5.5%)	
Kidney	3 (4.8%)	14 (4.9%)	17 (4.9%)	
Stomach	4 (6.5%)	10 (3.5%)	14 (4.1%)	
Lymphoid	2 (3.2%)	11 (3.9%)	13 (3.8%)	
Pleura	3 (4.8%)	7 (2.5%)	10 (2.9%)	
Head and neck	0	6 (2.1%)	6 (1.7%)	
Hematologic	0	5 (1.8%)	5 (1.4%)	
Liver	0	5 (1.8%)	5 (1.4%)	
Uterus	0	5 (1.8%)	5 (1.4%)	
Brain	2 (3.2%)	2 (0.7%)	4 (1.2%)	
Esophagus	0	4 (1.4%)	4 (1.2%)	
Breast	0	3 (1.1%)	3 (0.9%)	
Ovary	0	3 (1.1%)	3 (0.9%)	
Large intestine	0	1 (0.4%)	1 (0.3%)	
Oropharynx	0	1 (0.4%)	1 (0.3%)	
Prostate	1 (1.6%)	0	1 (0.3%)	
Unspecified	3 (4.8%)	3 (1.1%)	6 (1.7%)	
Missing	0	17 (6.0%)	17 (4.9%)	
Reporter type				
Consumer	10 (16.1%)	32 (11.3%)	42 (12.2%)	0.546
Physician	32 (51.6%)	137 (48.4%)	169 (49.0%)	
Pharmacist	5 (8.1%)	23 (8.1%)	28 (8.1%)	
Other healthcare professional	15 (24.2%)	91 (32.2%)	106 (30.7%)	

### 3.3 Analysis of the time to onset with ICIs-induced ITP

**FIGURE 5:**
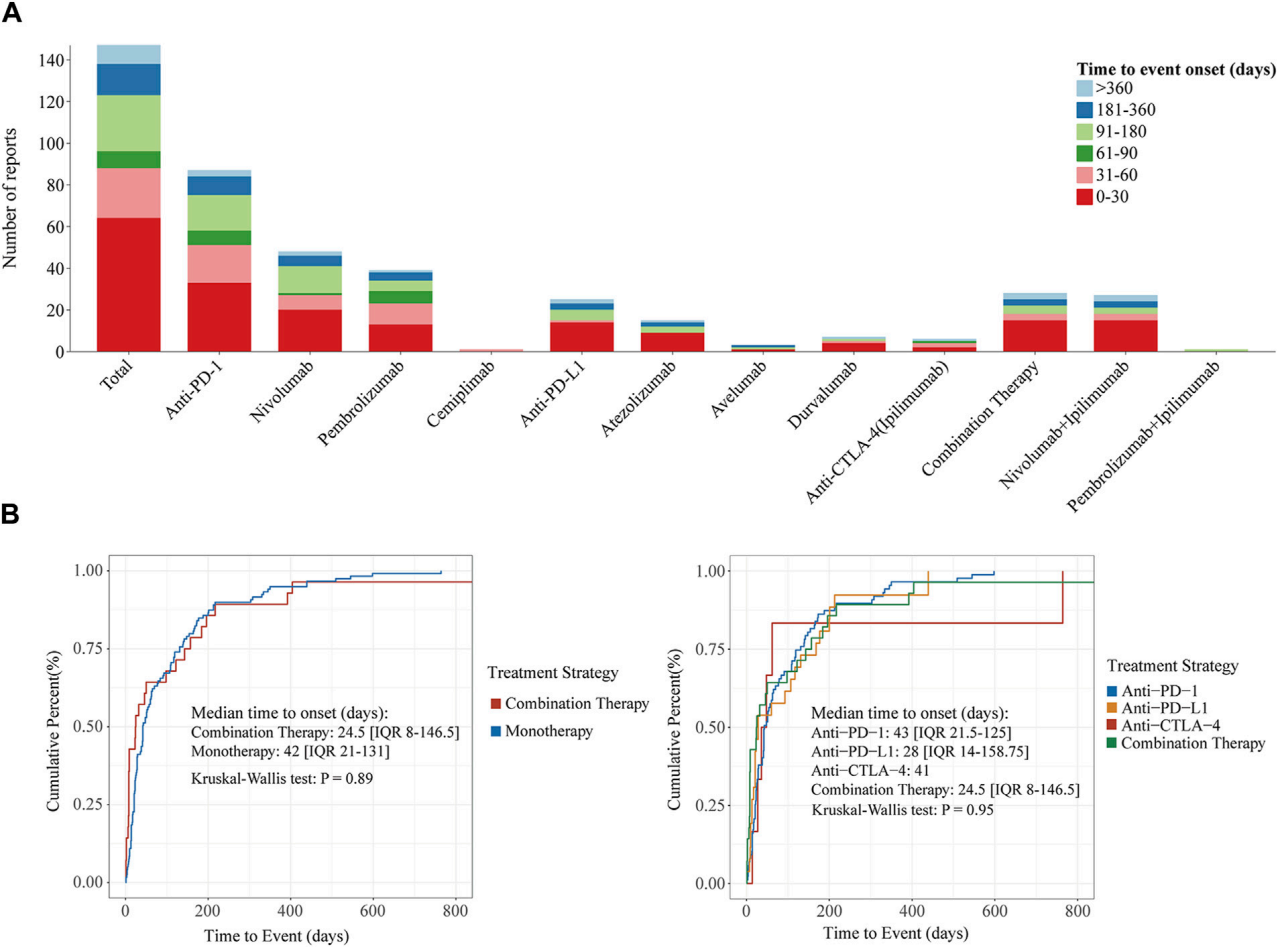
Time to onset of ICIs-induced ITP under different ICI treatment strategies. **(A)** Stacked plots of ICIs-induced ITP onset time under different ICI treatment strategies. The horizontal coordinate is the different treatment strategies and the vertical coordinate is the number of reports. Different colors represent different time to event onset. The columns are stacked by reports with different time to event onset. **(B)** Cumulative distribution curves of ICIs-induced ITP onset time under different ICI treatment strategies. From the left to right, the cumulative distribution curves demonstrate the onset time of ICIs-induced ITP after treatment with ICIs in different subgroups (combination Therapy vs. monotherapy and different ICI treatment strategies). Statistical tests were conducted using the Kruskal–Wallis test.

The ICIs-induced ITP report showed that the median time to ITP after treatment with ICIs was 42 days (IQR 17-135), and the majority of patients (64/147, 43.5%) developed ITP within 30 days of treatment with ICIs (Figure 5A). Compared to monotherapy, combination therapy had a significantly shorter median onset time (days: 24.5 vs. 42). In the monotherapy group, patients treated with anti-PD-L1 had the shortest median onset time of 28 days (IQR 14-158.75), whereas patients treated with anti-PD-1 and anti-CTLA-4 had a median onset time of greater than 1 month, 43 days (IQR 21.5–125) and 41 days. Notably, the median time to onset was the shortest in patients who received combination therapy at 24.5 days, followed by those treated with anti-PD-L1 at 28 days, both within 1 month. In contrast, patients treated with the anti-PD-1 antibody had the longest median time to disease onset at 43 days. In addition, log-rank tests showed (Figure 5B) that neither combination therapy and monotherapy (*p* = 0.89), multiple treatment strategies within the monotherapy group (*p* = 0.95), nor different ICI treatment strategies, influenced the median onset time of ICIs-induced ITP.

**FIGURE 6:**
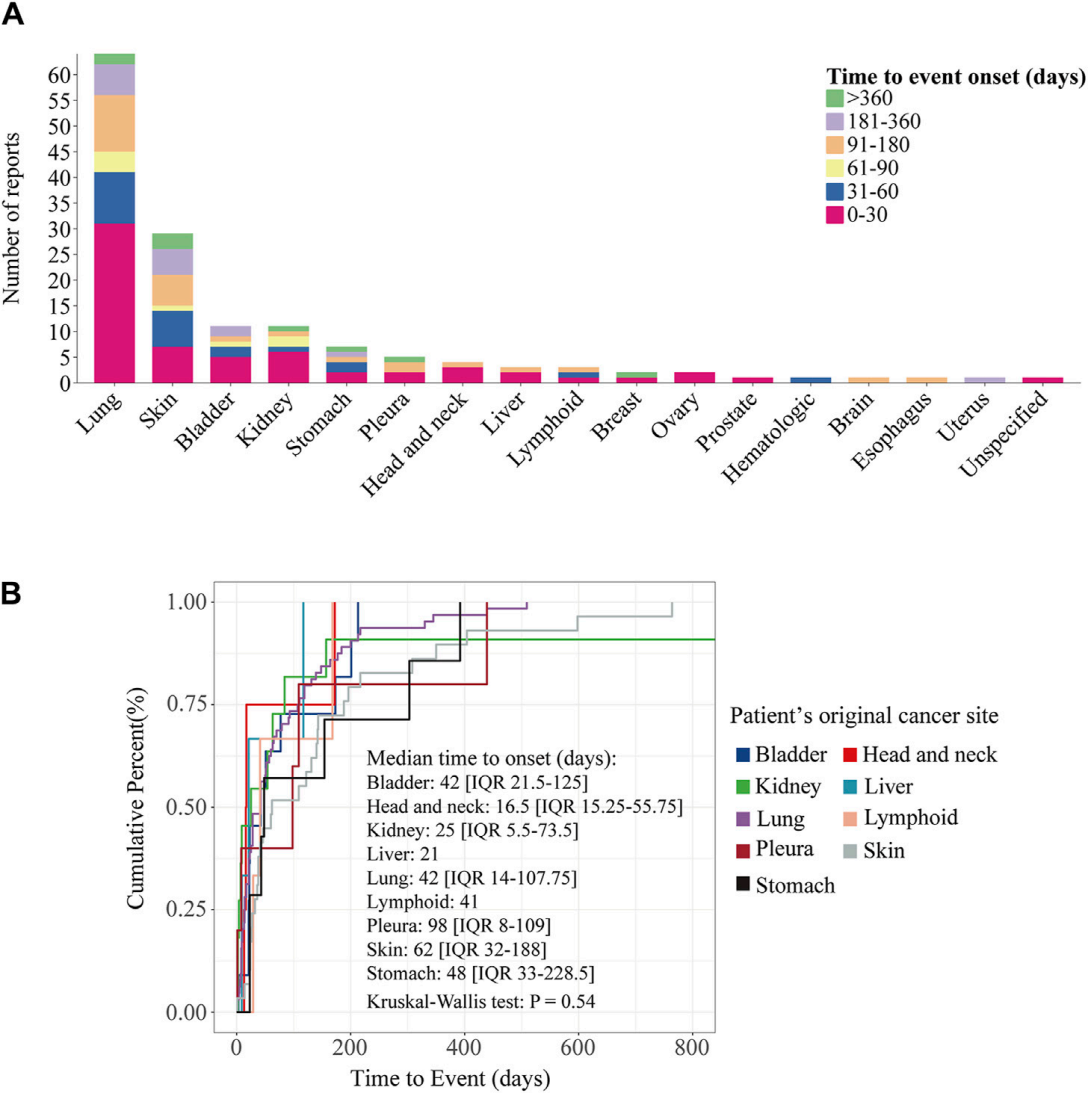
Time to onset of ICIs-induced ITP between different patient’s cancer original sites. **(A)** Stacked plots of ICIs-induced ITP onset time between different patient’s cancer original sites. The horizontal coordinate is the different patient’s cancer original sites and the vertical coordinate is the number of reports. Different colors represent different time to event onset. The columns are stacked by reports with different time to event onset. **(B)** Cumulative distribution curves of ICIs-induced ITP onset time between different patient’s cancer original sites. The horizontal coordinate is the time to onset (days) and the vertical coordinate is the cumulative percent. Statistical tests were conducted using the Kruskal–Wallis test.

When stratified by the original cancer sites of the patients, the results revealed that most patients with cancers originating from the lung (31/64, 48.4%), bladder (5/11, 45.5%), kidney (6/11, 54.5%), head and neck (3/4, 75%), and liver (2/3, 66.7%) experienced onset within 30 days. For patients with cancer originating from the skin (14/29, 48.3%), stomach (4/7, 57.1%), and lymphoid (2/3, 66.7%) the majority experienced onset within 60 days (Figure 6A). The top three original sites by median onset time were the head and neck (median onset time: 16.5, IQR 14-158.75), liver (median onset time: 21), and kidney (median onset time: 21, IQR 5.5-73.5). Log-rank test indicated no significant difference in the time to onset of ICI-induced ITP between among the different cancer original sites of patients (*p* = 0.54) (Figure 6B).

### 3.4 Analysis of the potential biological mechanism associated with ICIs-induced ITP

**FIGURE 7:**
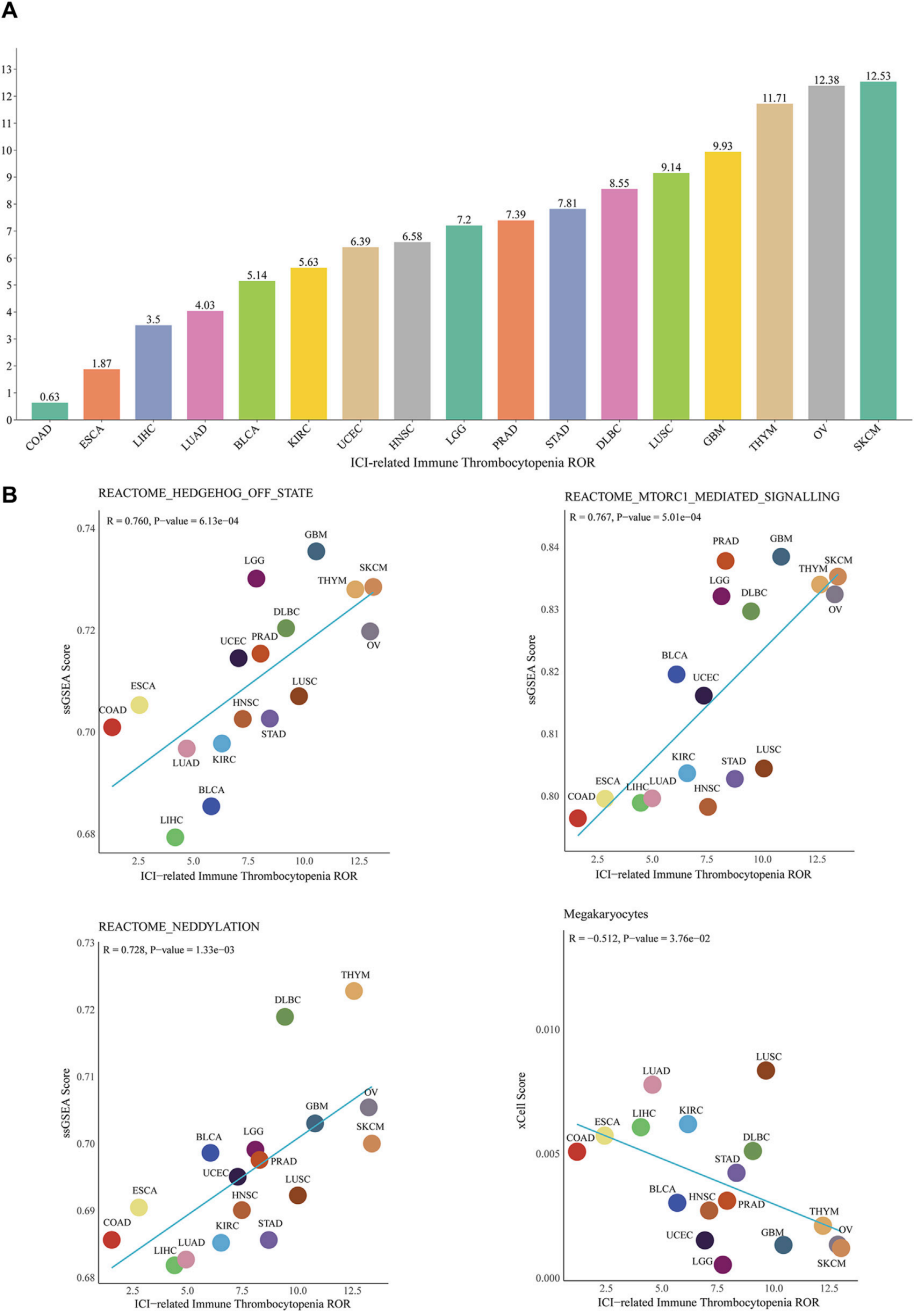
Correlation between ICIs-induced ITP and pan-cancer biological features. **(A)** ROR of ICIs-induced ITP in 17 cancer types. **(B)** The correlation analysis between the ROR of ICIs-induced ITP and ssGSEA enrichment scores for the mTOR, Hedgehog, and Neddylation signaling pathways, as well as megakaryocytes. SKCM: Skin Cutaneous Melanoma; LUSC: Lung squamous cell carcinoma; DLBC: Lymphoid Neoplasm Diffuse Large B-cell Lymphoma; STAD: Stomach adenocarcinoma; PRAD: Prostate adenocarcinoma; LGG: Brain Lower Grade Glioma; UCEC: Uterine Corpus Endometrial Carcinoma; LUAD: Lung adenocarcinoma; LIHC: Liver hepatocellular carcinoma; ESCA: Esophageal carcinoma; COAD: Colon adenocarcinoma.

In addition, the results of conjoint analysis showed that at the pan-cancer level, the top three cancers with ROR for ICIs-induced ITP were skin cutaneous melanoma (12.5, 95% CI 5.8-27.2), ovarian serous cystadenocarcinoma (12.4, 95% CI 3.6-42.8) and thymoma (11.7, 95% CI 2.8-49.0), with the lowest ROR in Colon adenocarcinoma (0.6, 95% CI 0.09-4.5) (Figure 7A). The correlation analysis of the potential mechanism mining and TCGA database showed that the pan-cancer level ICIs-induced ITP ROR was significantly and positively correlated with the mTORC1 (R = 0.767, *p* < 0.001), Hedgehog (R = 0.76, *p* < 0.001), and neddylation signaling pathways (R = 0.728, *p* < 0.01) and significantly negatively correlated with megakaryocytes (R = −0.512, *p* < 0.05) (Figure 7B).

## 4 Discussion

To our knowledge, this is the first pharmacovigilance and potential mechanism mining analysis of ICIs-induced ITP based on the FAERS database with the TCGA pan-canceromics database. In this study, we first performed an ICIs-induced ITP comprehensive pharmacovigilance analysis based on complete 11-year-long data from 2012 to 2022 in the FAERS database, performed a disproportionality analysis for ICIs-induced ITP reports, and described the clinical characteristics of the reported cases. The potential biological mechanisms of ICIs-induced ITP were explored at the level of biological signaling pathways and cellular infiltration in combination with TCGA pan-cancer transcriptomic data.

With the full promotion of ICIs in tumor therapy in the past decade, their multi-organ drug toxicity caused by them has gradually attracted attention. Several rare irAEs have also gradually emerged, including hem-irAEs are one of them, with an incidence of approximately 0.04–3.6% among all irAEs (Kramer et al., 2021). The prevalence of ITP, the most commonly reported hem-irAEs, is approximately 0.2–2.8% of all hem-irAEs (Haddad et al., 2022). Previous research has indicated that ITP occurs in 29% of 63 cases of hem-irAEs (Michot et al., 2019). The results of this study showed that the number of ICIs-induced ITP cases increased annually from 2012 to 2022; however, the maximum number of cases reported was only 73, and the proportion of ICIs-induced ITP among all irAEs ranged from 0.09 to 0.41%, which showed that with the promotion of ICIs, the number of ICIs-induced ITP cases has increased but is still relatively rare.

Seven ICI monotherapy and two ICI combination therapy patient adverse reaction reports were included. Notably, all ICI treatment strategies showed significant signals when up to four algorithms were used for adverse reaction signal mining. This indicates a significant correlation between different ICI treatment strategies and ICIs-induced ITP, which deserves clinicians’ attention. Among the ICI combination regimens included in this study, pembrolizumab in combination with ipilimumab had the highest ROR (14.9, 95%CI 3.7-59.9), while the ROR (10, 95% CI 7.6-13.1) for nivolumab in combination with ipilimumab was higher than that for the vast majority of ICI monotherapy, which may indicate that patients treated with anti-PD-1 in combination with anti-CTLA-4 are more likely to be at increased risk of ICIs-induced ITP. This is also the first time that a pharmacovigilance perspective has been presented using large-scale adverse event data. Then among ICI monotherapy regimens, the highest number of case reports and ROR were reported for those treated with anti-PD-1 (7.4 95%CI 6.5-8.4), followed by anti-PD-L1 (6, 95%CI 4.4-8.2) and anti-CTLA-4 (4, 95%CI 2.6-6.3), which may indicate that anti-PD-1 significantly increases the risk of ITP (Ohashi et al., 2023). In addition, when all 9 ICI treatment strategies were considered, avelumab had the highest ROR (14.6, 95% CI 6.5-32.5). This illustrates the strong correlation between avelumab and ITP, as Dorothea et al. reported a case of a male patient with metastatic Merkel cell carcinoma diagnosed with ITP 1 month after treatment with avelumab (10 mg/kg every 2 weeks). The patient died after 3 months of treatment despite multiple therapies, including corticosteroids, intravenous immunoglobulin, and methylprednisolone (Kratzsch et al., 2019).

In addition, this study described the clinical characteristics of the 345 ICIs-induced ITP cases included, and some similarities were observed when compared with published retrospective studies. The median age of the patients in this study was 69 years (IQR 60-76), which was similar to the results of another retrospective study (Davis et al., 2019). Patients with ICIs-induced ITP were predominantly male (61.2%), and the indications were mainly lung, skin, and urological tumors, which is consistent with the results of several previous retrospective studies. Davis et al. described the clinical characteristics of patients with ICIs-induced hematological toxicities based on VigiBase, the World Health Organization’s pharmacovigilance database for individual case safety reporting of adverse drug reactions, which included 168 cases of ICIs-induced hem-irAEs. The median age of 57 patients with ICIs-induced ITP was 65 years, and 58% of the patients were male (Davis et al., 2019). A retrospective analysis of 1, 038 patients treated with ICIs at the Ohio State University Comprehensive Cancer Center between January 2011 and June 2017 by Haddad et al. found that 28 patients with ICIs-induced ITP were 55.6% male (Haddad et al., 2022). Moore et al. reviewed 57 case reports of patients with ICIs-induced ITP from previous studies, of which 60% were male (Moore et al., 2024). Interestingly, the primary indications for patients with ICIs-induced ITP in all three studies were lung cancer, skin cancer, and urological tumors. Several observational studies have described the time of ITP onset in patients with ICIs-induced ITP. Michot et al. reviewed 63 case reports of patients with ICIs-induced hem-irAEs, of which 18 patients with ICIs-induced ITP had a median time to onset of 42 days, which is consistent with the results of this study (Michot et al., 2019). Additionally, several retrospective studies have been shown that the median time to onset in patients with ICIs-induced ITP ≤60 days (Davis et al., 2019; Martin et al., 2022; Moore et al., 2024), which emphasizes the need for close monitoring of the risk of patients experiencing ICIs-induced ITP within 60 days. Furthermore, the results of this study showed that the median time to onset in patients receiving combination therapy was 24.5 days, which was 17.5 days shorter than that in patients receiving monotherapy. In a case report by Mullally et al., a male patient with metastatic melanoma was diagnosed with ITP after anemia occurred 11 days after treatment with nivolumab in combination with ipilimumab. The patient died on the 15th day (Mullally et al., 2022). These results indicate that patients receiving combination therapy with anti-PD-1/L1 + anti-CTLA-4 should be closely monitored for the risk of morbidity and mortality for a shorter time (within 30 days). In addition, when stratified by the original cancer sites of patients, most patients cancers originating from the lung, head and neck, kidney, bladder, and liver had an onset time of less than 30 days, whereas most of patients with cancers originating from the skin, stomach, and lymphoid had an onset time of less than 60 days. Xie et al. (2021) reviewed 16 cases of lung cancer with ICI-induced ITP, of whom 6 (37.5%) patients experienced onset within 30 days. In the case review by Liu et al. (2020), 7 (63.6%) of 11 cases of skin melanoma with ICI-induced ITP experienced onset within 60 days. Although there was no significant difference in the time to onset of ICI-induced ITP among patients with different cancer original sites, the results of the present study still indicate the importance of closely monitoring the occurrence of ICI-induced ITP in patients with lung cancer and melanoma within 60 days. In the previous study, 62 (18%) of 345 patients with ICIs-induced ITP died, compared with a mortality rate of 5.6–11% in previous studies (Davis et al., 2019; Haddad et al., 2022; Moore et al., 2024). These results indicate that although ICI-induced ITP is relatively rare, severe cases can be life-threatening (Hasegawa et al., 2019). Therefore, clinicians should be aware of the severity of these adverse events.

Owing to the rarity of ICIs-induced ITP, previous studies and related descriptions are scarce, and the potential mechanism remains unclear. Therefore, we combined TCGA pan-cancer transcriptomic data for a preliminary exploration of the potential mechanism of ICIs-induced ITP. The results indicated that cutaneous melanoma exhibited the highest ICI-induced ITP ROR, which is consistent with the results described in several observational studies. Previous research has consistently demonstrated that the tumor type with the highest number of ICI-induced ITPs is melanoma, with other frequently reported tumor types including lung cancer, lymphoma, and renal cancer (Delanoy et al., 2019; Liu et al., 2020; Haddad et al., 2022). The occurrence of this phenomenon may be related to the population of patients with different tumor types and the indications for ICI treatment. Notably, PD-L1 overexpression has been reported on platelets of patients with lung cancer, which may result direct inhibition of platelets by anti-PD-L1 drugs (Rolfes et al., 2018). However, there are no reports revealing a specific mechanism for the increased likelihood of ICI-induced ITP in patients with melanoma. In this study, the enrichment of biological signaling pathways and cellular infiltration levels in pan-cancer correlated with ICIs-induced ITP ROR. The results showed that the pan-cancer level ICIs-induced ITP ROR was significantly positively correlated with the mTORC1, hedgehog, and neddylation signaling pathways, with a correlation coefficient >0.7, whereas it was significantly negatively correlated with the enrichment score of megakaryocytes. mTOR is an atypical serine/threonine (S/T) protein kinase, a member of the PI3K family, and its name is derived from its inhibitor, rapamycin (Jhanwar-Uniyal et al., 2024). Dysregulation of the mTOR-related signaling pathway leads to aberrant cell proliferation, migration, and survival and has been associated with the pathogenesis of several diseases (Chan, 2004; Laplante and Sabatini, 2012; Saxton and Sabatini, 2017; Panwar et al., 2023). Chen et al. recruited 190 ITP patients. Platelets from patients expressed significantly higher levels of mTOR and its phosphorylated proteins than healthy controls, indicating aberrant activation of mTOR pathways. (Chen et al., 2022). In previous studies, the mTOR inhibitor rapamycin was reported to reduce drug toxicity and reverse treatment resistance in combination with ICIs (Beeram et al., 2007; Esfahani et al., 2019), showing therapeutic potential against ICIs-induced ITP. Xing et al. reported the case of a 55-year-old patient with diffuse large B-cell lymphoma who developed severe thrombocytopenia after CAR-T cell therapy and whose platelet levels returned to normal on day 25 after treatment with rapamycin (Xing et al., 2021). Feng et al. recruited 86 patients with refractory/relapsed ITP who received rapamycin and showed that the overall response rate reached 85% after 3 months of therapy, indicating that rapamycin may be effective in treating refractory/relapsed ITP (Feng et al., 2020). Dysregulation of both the Hedgehog and Neddylation signaling pathways has been closely associated with multiple processes of tumor progression (Xu et al., 2022; Chen et al., 2023), however no relationship with ICIs-induced ITP has been reported, indicating that the potential mechanisms remain to be further explored. In addition, the pan-cancer levels of ICIs-induced ITP ROR were significantly negatively correlated with megakaryocytes, indicating that ICIs-induced ITP may be associated with megakaryocyte dysfunction. This mechanism may be due to the activation of immune-related genes and pathways during hematopoiesis, leading to the dysfunction of megakaryocyte progenitor cell differentiation into platelets (Liu et al., 2022). Notably, Sun et al. reported that megakaryocyte dysfunction in ITP is associated with abnormal autophagy, which could be due to the deletion of autophagy-related genes, such as ATG7, and the overactivation of mTOR-related signaling pathways (Sun and Shan, 2019), which is highly relevant to the results of our study. Currently, there is no specific treatment for patients with ICIs-induced ITP, usually when Grade ≥3, ICIs treatment is discontinued and treatment regimens such as corticosteroids, intravenous immunoglobulin, and rituximab are recommended, however, the above treatment regimens do not result in long-term remission (Marini et al., 2022). The results of our study indicate that the potential mechanism of ICIs-induced ITP may be highly related to megakaryocyte autophagy dysfunction induced by the overactivation of the mTOR-related signaling pathway. Drugs related to this mechanism, such as rapamycin and decitabine, may also provide novel regimens for the clinical management and treatment of patients with ICIs-induced ITP. In conclusion, the mechanisms underlying ICIs-induced ITP require further investigation and validation.

This study had some limitations. First, the FAERS database is a global spontaneous reporting system with some information bias owing to different levels of awareness among reporters and differences in drug markets. Secondly, we were unable to confirm a causal relationship between ICIs and ICIs-induced ITP because of the confounding effects of multiple variables. In addition, because the FAERS database does not contain all global case reports of patients with ICIs-induced ITP, we were unable to determine the prevalence of ICIs-induced ITP in a wider population. Finally, this study, as a large-scale pharmacovigilance and mechanistic exploratory analysis, was based on the data level, and all results of this study require confirmation in large-scale prospective and basic research.

## 5 Conclusion

In conclusion, the prevalence of ICIs-induced ITP was 0.09–0.41% of irAEs in the FAERS database (2012—2022). In addition to tremelimumab, there were significant reporting signals for ITP with ICI monotherapy and combination therapy (nivolumab/ipilimumab and pembrolizumab/ipilimumab). Patients treated with anti-PD-1 in combination with anti-CTLA-4 are more likely to have an increased risk of ICIs-induced ITP. Therefore, careful monitoring of patients receiving ICI combination therapy and ICI monotherapy for their risk of ITP occurrence within 30 days and 60 days, respectively, is recommended. In addition, patients with cutaneous melanoma are at a higher risk of developing ITP when treated with ICI and should be closely monitored for this risk within 60 days of treatment. The potential mechanism of ICIs-induced ITP may be related to autophagic dysfunction of megakaryocytes induced by overactivation of the mTOR-related signaling pathway. The results of this study provide a comprehensive understanding of ICIs-induced ITP, and clinicians should consider this potentially fatal adverse effect.

## Data Availability

Publicly available datasets were analyzed in this study. This data can be found here: FAERS data: https://fis.fda.gov/extensions/FPD-QDE-FAERS/FPD-QDE-FAERS.html; TCGA data: https://portal.gdc.cancer.gov/v1/annotations.

## References

[B1] Aran D. Hu Z. Butte A. J. (2017). xCell: digitally portraying the tissue cellular heterogeneity landscape. Genome Biol. 18, 220. 10.1186/s13059-017-1349-1 29141660 PMC5688663

[B2] Beeram M. Tan Q.-T. N. Tekmal R. R. Russell D. Middleton A. DeGraffenried L. A. (2007). Akt-induced endocrine therapy resistance is reversed by inhibition of mTOR signaling. Ann. Oncol. 18, 1323–1328. 10.1093/annonc/mdm170 17693645

[B3] Cancer Genome Atlas Research Network Weinstein J. N. Collisson E. A. Mills G. B. Shaw K. R. M. Ozenberger B. A. Ellrott K. (2013). The cancer genome Atlas pan-cancer analysis project. Nat. Genet. 45, 1113–1120. 10.1038/ng.2764 24071849 PMC3919969

[B4] Chan S. (2004). Targeting the mammalian target of rapamycin (mTOR): a new approach to treating cancer. Br. J. Cancer 91, 1420–1424. 10.1038/sj.bjc.6602162 15365568 PMC2409926

[B5] Chen S. Zhou B. Huang W. Li Q. Yu Y. Kuang X. (2023). The deubiquitinating enzyme USP44 suppresses hepatocellular carcinoma progression by inhibiting Hedgehog signaling and PDL1 expression. Cell Death Dis. 14, 830. 10.1038/s41419-023-06358-y 38097536 PMC10721641

[B6] Chen Y. Luo L. Zheng Y. Zheng Q. Zhang N. Gan D. (2022). Association of platelet desialylation and circulating follicular helper T cells in patients with thrombocytopenia. Front. Immunol. 13, 810620. 10.3389/fimmu.2022.810620 35450072 PMC9016750

[B7] Davis E. J. Salem J.-E. Young A. Green J. R. Ferrell P. B. Ancell K. K. (2019). Hematologic complications of immune checkpoint inhibitors. Oncologist 24, 584–588. 10.1634/theoncologist.2018-0574 30819785 PMC6516131

[B8] Delanoy N. Michot J.-M. Comont T. Kramkimel N. Lazarovici J. Dupont R. (2019). Haematological immune-related adverse events induced by anti-PD-1 or anti-PD-L1 immunotherapy: a descriptive observational study. Lancet Haematol. 6, e48–e57. 10.1016/S2352-3026(18)30175-3 30528137

[B9] Esfahani K. Al-Aubodah T.-A. Thebault P. Lapointe R. Hudson M. Johnson N. A. (2019). Targeting the mTOR pathway uncouples the efficacy and toxicity of PD-1 blockade in renal transplantation. Nat. Commun. 10, 4712. 10.1038/s41467-019-12628-1 31624262 PMC6797722

[B10] Feng Y. Xiao Y. Yan H. Wang P. Zhu W. Cassady K. (2020). Sirolimus as rescue therapy for refractory/relapsed immune thrombocytopenia: results of a single-center, prospective, single-arm study. Front. Med. (Lausanne) 7, 110. 10.3389/fmed.2020.00110 32296709 PMC7136762

[B11] Guo Q. Zhao J. N. Liu T. Gao J. Guo H. Cheng J. M. (2023). Immune checkpoint inhibitor-induced aplastic anaemia: case series and large-scale pharmacovigilance analysis. Front. Pharmacol. 14, 1057134. 10.3389/fphar.2023.1057134 36778017 PMC9908595

[B12] Haddad T. C. Zhao S. Li M. Patel S. H. Johns A. Grogan M. (2022). Immune checkpoint inhibitor-related thrombocytopenia: incidence, risk factors and effect on survival. Cancer Immunol. Immunother. 71, 1157–1165. 10.1007/s00262-021-03068-2 34618180 PMC9015999

[B13] Hasegawa T. Ozaki Y. Inoue T. Watanabe Y. Fukuhara M. Yamaura T. (2019). Nivolumab-related severe thrombocytopenia in a patient with relapsed lung adenocarcinoma: a case report and review of the literature. J. Med. Case Rep. 13, 316. 10.1186/s13256-019-2245-y 31647029 PMC6813076

[B14] Jhanwar-Uniyal M. Zeller S. L. Spirollari E. Das M. Hanft S. J. Gandhi C. D. (2024). Discrete mechanistic target of rapamycin signaling pathways, stem cells, and therapeutic targets. Cells 13, 409. 10.3390/cells13050409 38474373 PMC10930964

[B15] Kramer R. Zaremba A. Moreira A. Ugurel S. Johnson D. B. Hassel J. C. (2021). Hematological immune related adverse events after treatment with immune checkpoint inhibitors. Eur. J. Cancer 147, 170–181. 10.1016/j.ejca.2021.01.013 33706206

[B16] Kratzsch D. Simon J.-C. Pönitzsch I. Ziemer M. (2019). Lethal thrombocytopenia in a patient treated with avelumab for metastatic Merkel cell carcinoma. J. Dtsch. Dermatol Ges. 17, 73–75. 10.1111/ddg.13722 30549433

[B17] Kroll M. H. Rojas-Hernandez C. Yee C. (2022). Hematologic complications of immune checkpoint inhibitors. Blood 139, 3594–3604. 10.1182/blood.2020009016 34610113 PMC9227102

[B18] Laplante M. Sabatini D. M. (2012). mTOR signaling in growth control and disease. Cell 149, 274–293. 10.1016/j.cell.2012.03.017 22500797 PMC3331679

[B19] Li J. Deng X. Wang B. Li W. (2019). Fatal outcome of atezolizumab in a patient with immune-mediated pneumonitis, thrombocytopenia, and cardiac dysfunction: a case report. Int. J. Clin. Pharmacol. Ther. 57, 607–611. 10.5414/CP203448 31488241

[B20] Liu X. Liang X. Liang J. Li Y. Wang J. (2020). Immune thrombocytopenia induced by immune checkpoint inhibitors in solid cancer: case report and literature review. Front. Oncol. 10, 530478. 10.3389/fonc.2020.530478 33365266 PMC7750527

[B21] Liu Y. Zuo X. Chen P. Hu X. Sheng Z. Liu A. (2022). Deciphering transcriptome alterations in bone marrow hematopoiesis at single-cell resolution in immune thrombocytopenia. Signal Transduct. Target Ther. 7, 347. 10.1038/s41392-022-01167-9 36202780 PMC9537316

[B22] Marei H. E. Hasan A. Pozzoli G. Cenciarelli C. (2023). Cancer immunotherapy with immune checkpoint inhibitors (ICIs): potential, mechanisms of resistance, and strategies for reinvigorating T cell responsiveness when resistance is acquired. Cancer Cell Int. 23, 64. 10.1186/s12935-023-02902-0 37038154 PMC10088229

[B23] Marini I. Uzun G. Jamal K. Bakchoul T. (2022). Treatment of drug-induced immune thrombocytopenias. Haematologica 107, 1264–1277. 10.3324/haematol.2021.279484 35642486 PMC9152960

[B24] Martin M. Nguyen H.-M. Beuvon C. Bene J. Palassin P. Atzenhoffer M. (2022). Immune checkpoint inhibitor-related cytopenias: about 68 cases from the French pharmacovigilance database. Cancers (Basel) 14, 5030. 10.3390/cancers14205030 36291814 PMC9599380

[B25] Michot J. M. Lazarovici J. Tieu A. Champiat S. Voisin A. L. Ebbo M. (2019). Haematological immune-related adverse events with immune checkpoint inhibitors, how to manage? Eur. J. Cancer 122, 72–90. 10.1016/j.ejca.2019.07.014 31634647

[B26] Moore D. C. Elmes J. B. Arnall J. R. Strassel S. A. Patel J. N. (2024). PD-1/PD-L1 inhibitor-induced immune thrombocytopenia: a pharmacovigilance study and systematic review. Int. Immunopharmacol. 129, 111606. 10.1016/j.intimp.2024.111606 38359661

[B27] Mullally W. J. Cooke F. J. Crosbie I. M. Kumar S. Abernethy V. E. Jordan E. J. (2022). Case report: thrombotic-thrombocytopenic purpura following ipilimumab and nivolumab combination immunotherapy for metastatic melanoma. Front. Immunol. 13, 871217. 10.3389/fimmu.2022.871217 35514990 PMC9067158

[B28] Ohashi T. Takase-Minegishi K. Maeda A. Hamada N. Yoshimi R. Kirino Y. (2023). Incidence and risk of hematological adverse events associated with immune checkpoint inhibitors: a systematic literature review and meta-analysis. J. Hematol. 12, 66–74. 10.14740/jh1090 37187501 PMC10181326

[B29] Panwar V. Singh A. Bhatt M. Tonk R. K. Azizov S. Raza A. S. (2023). Multifaceted role of mTOR (mammalian target of rapamycin) signaling pathway in human health and disease. Signal Transduct. Target Ther. 8, 375. 10.1038/s41392-023-01608-z 37779156 PMC10543444

[B30] Ramos-Casals M. Brahmer J. R. Callahan M. K. Flores-Chávez A. Keegan N. Khamashta M. A. (2020). Immune-related adverse events of checkpoint inhibitors. Nat. Rev. Dis. Prim. 6, 38. 10.1038/s41572-020-0160-6 32382051 PMC9728094

[B31] Rolfes V. Idel C. Pries R. Plötze-Martin K. Habermann J. Gemoll T. (2018). PD-L1 is expressed on human platelets and is affected by immune checkpoint therapy. Oncotarget 9, 27460–27470. 10.18632/oncotarget.25446 29937998 PMC6007942

[B32] Saxton R. A. Sabatini D. M. (2017). mTOR signaling in growth, metabolism, and disease. Cell 168, 960–976. 10.1016/j.cell.2017.02.004 28283069 PMC5394987

[B33] Subramanian A. Tamayo P. Mootha V. K. Mukherjee S. Ebert B. L. Gillette M. A. (2005). Gene set enrichment analysis: a knowledge-based approach for interpreting genome-wide expression profiles. Proc. Natl. Acad. Sci. U. S. A. 102, 15545–15550. 10.1073/pnas.0506580102 16199517 PMC1239896

[B34] Sun R.-J. Shan N.-N. (2019). Megakaryocytic dysfunction in immune thrombocytopenia is linked to autophagy. Cancer Cell Int. 19, 59. 10.1186/s12935-019-0779-0 30923461 PMC6419848

[B35] Xie W. Hu N. Cao L. (2021). Immune thrombocytopenia induced by immune checkpoint inhibitrs in lung cancer: case report and literature review. Front. Immunol. 12, 790051. 10.3389/fimmu.2021.790051 34956221 PMC8695900

[B36] Xing L. Wang Y. Liu H. Gao S. Shao Q. Yue L. (2021). Case report: sirolimus alleviates persistent cytopenia after CD19 CAR-T-cell therapy. Front. Oncol. 11, 798352. 10.3389/fonc.2021.798352 35004324 PMC8733571

[B37] Xu T. Ma Q. Li Y. Yu Q. Pan P. Zheng Y. (2022). A small molecule inhibitor of the UBE2F-CRL5 axis induces apoptosis and radiosensitization in lung cancer. Signal Transduct. Target Ther. 7, 354. 10.1038/s41392-022-01182-w 36253371 PMC9576757

[B38] Zhou C. Peng S. Lin A. Jiang A. Peng Y. Gu T. (2023). Psychiatric disorders associated with immune checkpoint inhibitors: a pharmacovigilance analysis of the FDA Adverse Event Reporting System (FAERS) database. EClinicalMedicine 59, 101967. 10.1016/j.eclinm.2023.101967 37131541 PMC10149185

